# Cystic and Fatty Tumor of the Rectum

**Published:** 1872-05

**Authors:** W. D. Hoyt

**Affiliations:** Rome, Georgia


### CYSTIC AND FATTY TUMOR OF THE RECTUM.

REPORTED BY W. D. HOYT, M.D.. ROME, GEORGIA.

[Contributed to the Atlanta Academy of Medicine.]

On the 7th of May, 1871, I was called in great haste to see Caroline H——, her husband, who came for me, telling me she had that morning passed a large tumor from the bowel. On arriving at the house, very sceptical about the diagnosis in the case, I found a dark mulatto woman, aged apparently about fifty-five — married, the mother of several children, quite emaciated, and presenting the appearance of much and prolonged suffering. She had been in feeble health for several years; had had great difficulty in evacuating her bowels, having only been able to obtain an action from the use of medicine, and in the recumbent position. There had been, at times, discharges of pus, and another physician had treated her, a few months before, for what he considered dysentery.

On examination, I found the tumor had indeed passed from the bowel. There it was — a pear-shaped tumor, as large as a child's head, covered with mucous membrane, attached to a pedicle about two inches broad and a third of an inch thick; this pedicle extended up the bowel further than I could reach with my finger. The day before I saw her, the tumor had descended to the anus, as if to protrude, but had been pushed back. This morning it had escaped from the bowel without any evacuation of its contents.

Preparing a long stout ligature, I threw it around the pedicle, drew it as tight as possible, and secured it. Then, having a woman hold the buttock as well as possible out of the way, I cut through the tumor, not being able to cut through the pedicle without endangering the slipping of the ligature. As soon as the size of the tumor was reduced by division, it was immediately withdrawn into the bowel, carrying with it several inches of the ligature, and pressing from it, as it passed the sphincter, some small clots of blood and lumps of pus and mucus. The patient complained much of pain in the abdomen — commencing first in the left pelvic region, extending thence to the right, and then over the whole of the abdomen. Opiates per os and anus, light nourishment, and turpentine stupes and poultices, constituted the treatment. Injections of water, thrown into the bowel with a female syringe (the sphincter being sufficiently enlarged as easily to allow its passage), returned unrestrainedly, bringing away some lumps of pus and mucus. The patient's extremities were cold, the pulse feeble, about ninety. She became easier under the influence of opium, but died about twelve hours after the removal of the tumor. No post-mortem allowed.

The tumor was covered with a thick mucous membrane, to which quantities of viscid mucus were adherent. On opening it, several cysts were apparent, filled with a clear liquid, and in several of them, deposits of fat were accumulating. At the base were some cysts completely filled with fat. One cyst was ruptured, and filled with clotted blood. There was a superficial ulcer, at the base of the tumor, about the size of a silver half dollar. A stout band, apparently of fibrous tissue, penetrated the tumor, from which it may have grown, and which, before its section, was very tense.

The portion of tumor removed with the cyst, emptied and stripped of fluid, weighed nine ounces. It measures now, after having laid in a strong solution of salt and water twenty days, five and a half inches in length, and from three and a third to three and a half inches in diameter at its base.

The symptoms seemed to me to indicate that death in this case was caused by peritonitis. There was no hemorrhage, so far as I could judge, and the pain complained of seemed characteristic of acute peritonitis. If this supposition is correct, the question arises, How was the peritonitis produced? It seemed not unlikely that the bowel in the neighborhood of the tumor was diseased, perhaps ulcerated, and that in the violent strain upon the tumor, indicated by its rapid return when its size was reduced, and caused by the tenesmus resulting from the larger end of a pear-shaped body passing first out of the sphincter, the bowel might have been torn, and peritonitis have been thus produced. Without this supposition, I am unable to explain the occurrence of death.

